# microRNA-145-5p inhibits prostate cancer bone metastatic by modulating the epithelial-mesenchymal transition

**DOI:** 10.3389/fonc.2022.988794

**Published:** 2022-09-06

**Authors:** Bingfeng Luo, Yuan Yuan, Yifei Zhu, Songwu Liang, Runan Dong, Jian Hou, Ping Li, Yaping Xing, Zhenquan Lu, Richard Lo, Guan-Ming Kuang

**Affiliations:** ^1^ Division of Urology, Department of Surgery, The University of Hong Kong-Shenzhen Hospital, Shenzhen, China; ^2^ Department of Pathology, The University of Hong Kong-Shenzhen Hospital, Shenzhen, China; ^3^ Department of Orthopedics and Traumatology, The University of Hong Kong-Shenzhen Hospital, Shenzhen, China

**Keywords:** miRNA-145-5p, prostate cancer bone metastasis, apoptosis, epithelial-mesenchymal transition, non-coding RNA

## Abstract

**Objective:**

To investigate the effects of miRNA-145-5p on the tumor development and progression of prostate cancer (Pca) bone metastasis.

**Methods:**

Levels of miRNA-145-5p were assessed by real-time quantitative PCR in PC3 (bone metastatic Pca cells), 22RV1 (non-metastatic Pca cells), RWPE-1 (non-cancerous prostate epithelial cells) and Pca tissues collected from patients with and without bone metastases. The impact of miRNA-145-5p on cell proliferation was tested by CCK8 assay, colony formation assay and flow cytometric cell cycle analysis. Effects on invasion and migration of PC3 cells were determined by Transwell and wound healing assays. Western blotting, enzyme-linked immunosorbent assay, and flow cytometry apoptosis analyses were also performed to assess roles in metastasis.

**Results:**

Levels of miRNA-145-5p were decreased in Pca bone metastases and miRNA-145-5p inhibited cell proliferation, migration and invasion. miRNA-145-5p inhibited the expression of basic fibroblast growth factor (bFGF), insulin-like growth factor (IGF) and transforming growth factor-β (TGF-β) in PC3 cells. miR-145-5p increased the expression of the epithelial marker E-cadherin and reduced the expression of matrix metalloproteinase 2 and 9 (MMP-2 and MMP-9). It was found that miRNA-145-5p mediated the epithelial-mesenchymal transition (EMT) and induced apoptosis.

**Conclusions:**

miRNA-145-5p negatively regulated the EMT, inhibited Pca bone metastasis and promoted apoptosis in Pca bone metastasis. Mimicry of miRNA-145-5p action raises the possibility of a novel target for treating Pca with bone metastases.

## Introduction

Prostate cancer (Pca) represents a supreme challenge to human health with 1.3 million global cases diagnosed annually and 400000 deaths from metastatic Pca (1). Bone is the most common site of Pca metastasis, rendering patients predisposed to adverse skeletal events, including bone pain, spinal cord compression, pathological fractures and poor prognosis (1, 2). Stephen Page was the first to address the complexity of bone and proposed in 1889 that cancer cells tend to migrate to organs where they may be “sown” into advantageous “soil” leading to the development of metastatic lesions. Clear distinctions among the “soils” of potential metastatic sites have become apparent, with metastases being common in pelvic bones, ribs, vertebrae and the termini of long bones (2, 3). Therefore, there is a pressing need to identify the genes responsible for Pca bone metastasis in order to facilitate treatment advances. Previous reports have demonstrated the involvement of osteonectin, vascular endothelial growth factor (VEGF), TGF-β and C-X-C motif chemokine ligand 12 (CXCL12) in the extravasation and direction of Pca cells to bone (3). Besides, cadherin 11 mediates the interaction between Pca cells and osteoblasts (4) and urokinase (N-terminal fragment) stimulates Pca proliferation (5). Tumor microenvironmental factors, such as IGF, bFGF and TGF-β regulate Pca cell survival and growth (6). The regulator of TGF-β signal transduction, prostate transmembrane protein androgen-induced 1 (PMEPA1), has an inhibitory effect on Pca bone metastasis (6). However, further exploration of potential clinical applications of these genes is required to assess implications for the treatment of Pca bone metastasis.

MicroRNAs (miRNAs) are defined as small noncoding RNAs and have attracted attention for their utility as clinical biomarkers of cancer and potential for novel cancer treatment targets (7, 8). They exert effects on gene expression which influence tumor development, progression and therapeutic responses, being involved in the osteomimicry, EMT and formation of osteoblasts and osteoclasts (9, 10). Indeed, dysregulation of the tumor suppressor or oncogene functions of miRNAs have been implicated in the onset of many cancers. Mimics of miRNAs and antagonists (antimiRNAs) look promising in preclinical development with some having entered clinical trials. A mimic of the tumor suppressor miRNA, miRNA-34, is currently in first stage clinical trials for cancer treatment (11). Stage 2 clinical trials of antimiRNA-s against miRNA-122 are being conducted for hepatitis patients (12). Ectopic expression of miRNA-203 in PC3, a Pca bone metastatic cell-line, has been shown to induce phenotypical morphology alterations from fibroblast-like to epithelial-like and suppress metastatic development in a mouse model of bone metastasis (13). This observation illustrates the importance of searching for miRNAs that are downregulated in Pca bone metastases and may constitute potential biomarkers for the occurrence of metastatic lesions. Moreover, the identification of downregulated miRNAs reveals the potential for the use of mimics as a new target in treating Pca bone metastasis.

## Materials and methods

### Patients

20 Pca patients at the University of Hong Kong-Shenzhen Hospital were enrolled in this study. Patients underwent pelvic magnetic resonance imaging (MRI), whole body bone scan or positron emission tomography-computed tomography (PET-CT) scan before prostate needle biopsy and immunohistochemical staining to confirm Pca diagnosis. Patients were allocated to one of two groups (n=10) according to the presence or absence of bone metastases. Bone metastasis was evaluated from a whole body bone scan or a PET-CT scan. All patients signed written informed consent for the use of Pca tissue specimens. Baseline information of the patient cohort is presented in **Table 1**. Approval was granted by the local Research Ethics Committee (No (2019).260) and the current study was performed in compliance with the Declaration of Helsinki (1964).

**Table 1:** Baseline characteristics of prostate cancer patients with and without bone metastases.

Variables	Prostate cancer with bone metastasis ( n=10)	Prostate cancer without bone metastasis ( n=10)	*F / Z*	*p value*
**Age (years)**				
$\bar{\text{x}}$ ±s	69.0±6.83	65.30±6.85	0.045	0.242
**BMI (kg/m^2^ )**				
$\bar{\text{x}}$ ±s	21.20±1.81	22.53±2.00	0.402	0.137
**tPSA (ng/mL)**				
$\bar{\text{x}}$ ±s	53.10±61.06	19.85±11.05	7.73	0.107
**Tumor stage, n (%)**				
** T1**	0 (0)	1(10%)	-2.1	0.036*
** T2**	2(20%)	4(40%)		
** T3**	4(40%)	5(50%)		
** T4**	3(30%)	0 (0)		
**Gleason score, n (%)**				
** ≤6**	1 (10%)	3 (30%)	-1.73	0.084
** 7**	2 (20%)	4 (40%)		
** ≤8**	7 (70%)	3 (30%)		
**Alkaline phosphatase**				
$\bar{\text{x}}$ ±s, (U/L)	106.20±59.12	106.30±25.59	3.276	0.996
**Serum Creatinine**				
$\bar{\text{x}}$ ±s, (umol/L)	87.90±15.26	85.90±14.95	0.131	0.771
**Glycosylated hemoglobin**				
$\bar{\text{x}}$ ±s, (%)	5.50±0.81	5.50±0.90	0.46	1

* indicates significant difference.

### RNA isolation and sequencing

TRIpure Total RNA Extraction Reagent (ELK Biotechnology, Wuhan, China) was used in accordance with the manufacturer’s instructions for isolation of total RNA from Pca tissues. Pellets containing RNA were rinsed with 75% ethanol, air-dried and resuspended in RNase inhibitor-containing RNase-free water (Thermo-Fisher-Scientific, Austria) for storage at -80°C. The concentration and purity of RNA were quantified by Agilent 2100 Bioanalyzer (Agilent Technologies Inc., USA) and sequencing libraries prepared using QIAseq miRNA Library Kit (QIAGEN, Germany) for sequencing with Illumina HiSeq2500. FastQC was employed to examine the quality of the microRNA sequencing reads by alignment with the human genome reference (GRCh38, Ensembl release 76) using FANSe3 with default parameter. miRNA expression was quantified with HTSeq v0.6.1p1 based on miRNA-base (https://www.miRNA-base.org/).

### Differential miRNA expression

Differential miRNA expression was analyzed by DESeq2 (version 1.20.0). The Benjamini-Hochberg approach allowed calculation of the false discovery rate (FDR)-adjusted p-values with multiple testing correction. FDR-adjusted p-values of < 0.01 and fold-change of > 2 were considered to indicate differential expression. miRNA target genes were predicted using the miRNA-TarBase database. KOBAS annotation was used for enrichment analyses, including Kyoto Encyclopedia of Genes and Genomes (KEGG) pathway and Gene Ontology (GO) analyses.

### Validation of miRNA by real-time quantitative PCR

Total RNA was extracted and used to synthesize cDNA using the EntiLink™ 1st Strand cDNA Synthesis Kit (ELK Biotechnology, Wuhan, China), by following the manufacturer’s instructions. RT-qPCR was conducted using EnTurbo™ SYBR Green PCR SuperMix (ELK Biotechnology). Relative gene expression levels of miRNA-145-5p were detected using U6 as the endogenous normalization control. All stem-loop primer sequences were as follows: hsa-miRNA-145-5p (MIMAT0000437): forward primer: CAGTTTTCCCAGGAATCCCT, reverse primer: CTCAACTGGTG TCGTGGAGTC; U6 (NR_004394.1): forward primer: CTCGCTTCGGCAGCACAT, reverse primer: AACGCTTCACGAATTTGCGT. Changes in miRNA expression were quantified using the double delta Ct equation.

### Cell culture and transfection

PC-3 (bone metastatic Pca cells) were cultured in DMEM (Gibco, USA) containing 10% FBS (Gibco) and penicillin-streptomycin and maintained at 5% CO_2_. Aliquots of 3×10^5^ cells were grown in 6-well plates and transfected with an hsa-miRNA-145-5p mimic or control (inactive) vector using Lipofectamine 2000 (Invitrogen, USA) by following the kit’s protocol.

### Cell proliferation and invasiveness

Cells were grown in a 96-well plate and cell numbers were counted using CCK-8 (Cell Counting Kit-8, Biosharp) with additional wound healing, colony formation and Transwell assays. Cell culture medium was supplemented with CCK8 reagent and absorbance was determined at 450 nm after incubation for 1 h. For the migration/wound healing assays, 3 × 10^5^ cells/well were grown in a 24-well plate, incubated for 16-18 h and cell monolayers scraped with a pipette tip to create a wound which was washed with PBS. After incubation for 24 h in culture medium, an inverted microscope with a digital camera was used to photograph wound closure. Colony formation was measured by resuspending the cells with 1ml medium and seeding a six-well plate with 500 cells per well. After 2 weeks, 6-well plates were fixed with paraformaldehyde (4%) for 30min at room temperature before washing with PBS, the addition of crystal violet staining and photographs taken under the microscope. Transwell assay was conducted to assess invasion. Briefly, cells (3×10^5^ cells/well) were grown in the upper Transwell chamber and incubated for 24 h at 37°C and 5% CO_2_. Cells were detached, rinsed with PBS and fixed with paraformaldehyde for 20 min. After washing, cells were stained with crystal violet (0.1%) for 10 min, rinsed with PBS and counted using a microscope (Olympus, Japan)

### Flow cytometry analysis

Flow cytometry-based apoptosis analysis was conducted by Annexin V-FITC cell apoptosis analysis kit (Sungen Biotech, Tianjin, China) and cell cycle analysis was carried out using a cell cycle analysis kit (YEASEN, Shanghai, China). Samples were visualized by FACSCalibur (BD Biosciences, USA) and data were analyzed with FlowJo software (Tree star Inc, CA).

### Enzyme linked immunosorbent assay

The cell culture medium was removed and centrifuged at 2000rpm for 20 minutes to remove impurities and cell fragments. Detection of human IGF, human TGF-β and human bFGF in the supernatant was performed by ELISA kits (Mlbio, Shanghai, China).

### Western blotting

Cellular protein was isolated with RIPA buffer and separated by SDS-PAGE with 10% Bis-Tris (Invitrogen, USA) before transfer onto nitrocellulose membranes. Membranes were blocked with a 5% solution of skimmed milk at 4°C for 1 h. Membranes were incubated with primary antibodies (Abcam, UK) raised against the following proteins: caspase 9 (ab115792), E-cadherin (ab1416), MMP2 (ab97779), MMP9 (ab137867) and GAPDH (ab8245) overnight at 4°C and rinsed for 20 min in Tween-TBS before incubation with secondary antibodies (1:10000, ASPEN, China). After a 20 min washing with T-TBS, proteins were visualized with an electroluminescence kit (ASPEN, Wuhan, China). The internal control was GAPDH.

### Statistical analysis

Means ± SD of three independent experiments were presented, and statistical analysis was conducted using GraphPad v4.1 (CA, USA). Data were compared between groups using a two-tailed unpaired Student’s t-test. A p-value of <0.05 was deemed statistically significant.

## Results

### Expression of miRNAs in Pca patients

**Figure 1:**
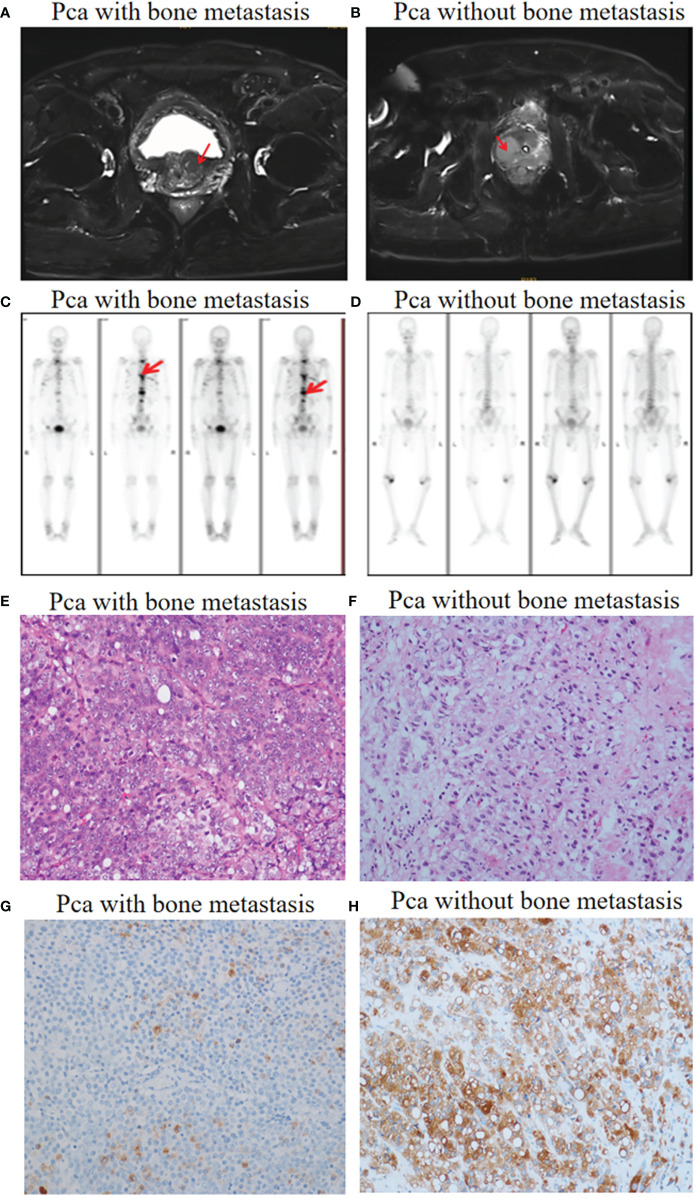
Pca with and without bone metastasis: diagnosis. **(A)** Pelvic MRI allowed visualization of a tumor in the prostate left lobe (red arrow). No metastases were found. **(B)** A tumor in the prostate right lobe with bone metastases (red arrow). **(C)** Whole body bone scanning showed Pca with bone metastases (red arrow). **(D)** Whole body bone scanning showed Pca without bone metastases. **(E, F)** HE staining images (objective 20X) showing prostate cancer morphology in the presence and absence of bone metastases. **(G, H)** Prostate-specific antigen (PSA) staining images (objective 20X) showing prostate cancer cells with flaky distribution in the presence and absence of bone metastases.

**Figure 2:**
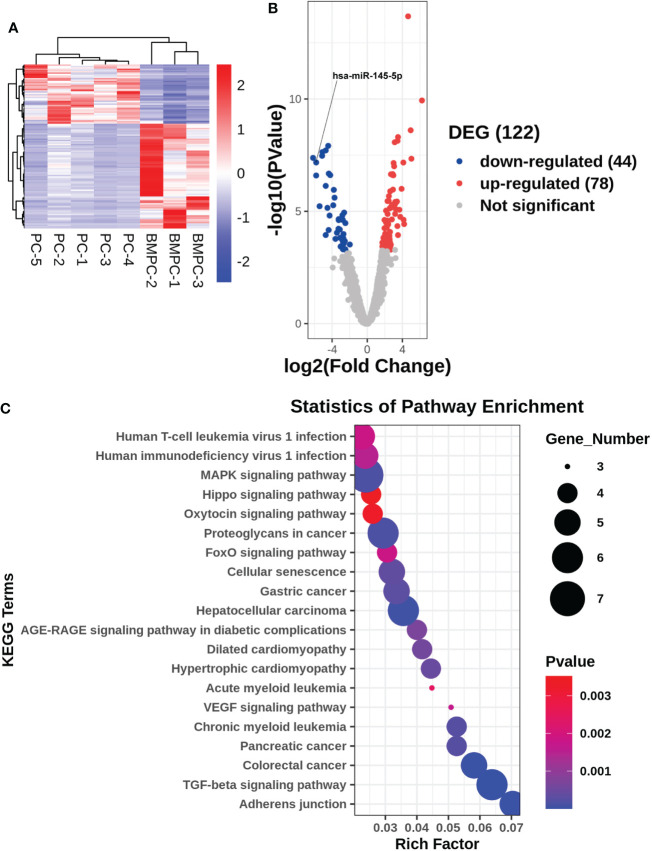
Gene expression profile for non-metastatic and bone metastatic prostate cancer. **(A, B)** Heat map and volcano plot showing miRNA expression levels. Right column: genes; red pixels: upregulated expression; blue pixels: downregulated expression. **(C)** KEGG pathway miRNA analysis. X axis: enriched genes; Y axis: KEGG pathway; dot color: p-value; dot size: gene number.

Patient diagnosis included a combination of pelvic magnetic resonance imaging (MRI), whole body bone scan or PET-CT scan, and histological analysis involving periodic acid-Schiff (PAS) and hematoxylin-eosin (HE) staining of tissue sections (**Figure 1**). High-throughput microRNA sequencing analysis was conducted on 5 Pca and 3 Pca bone metastatic samples. 122 microRNAs have been previously identified as having modulatory activity on Pca bone metastasis (**Figure 2A**). Of the set of 122 miRNAs, 78 (n=78) were upregulated and 44 downregulated in Pca with bone metastases compared to non-metastatic Pca (**Supplementary Data 1**). miRNA-145-5p was expressed at the one of the lowest levels in Pca tissues with bone metastasis with a false discovery rate>0.0001 (**Figure 2B**). miR-145-5p target genes were predicted using miRNA-TarBase (**Supplementary Data 2**). KEGG pathway analysis showed that the target genes of miR-145-5p exerted the regulatory activity over many tumor-related signaling pathways, including that of TGF-β and the cell-cell and cell-matrix adherens junction. (**Figure 2C**). Such target pathways have potential relevance concerning the metastasis of Pca to a bone site and indicate that miRNA-145-5p suppression may be responsible for the process.

### miRNA-145-5p suppressed proliferation of the Pca bone metastatic PC3 cells

**Figure 3:**
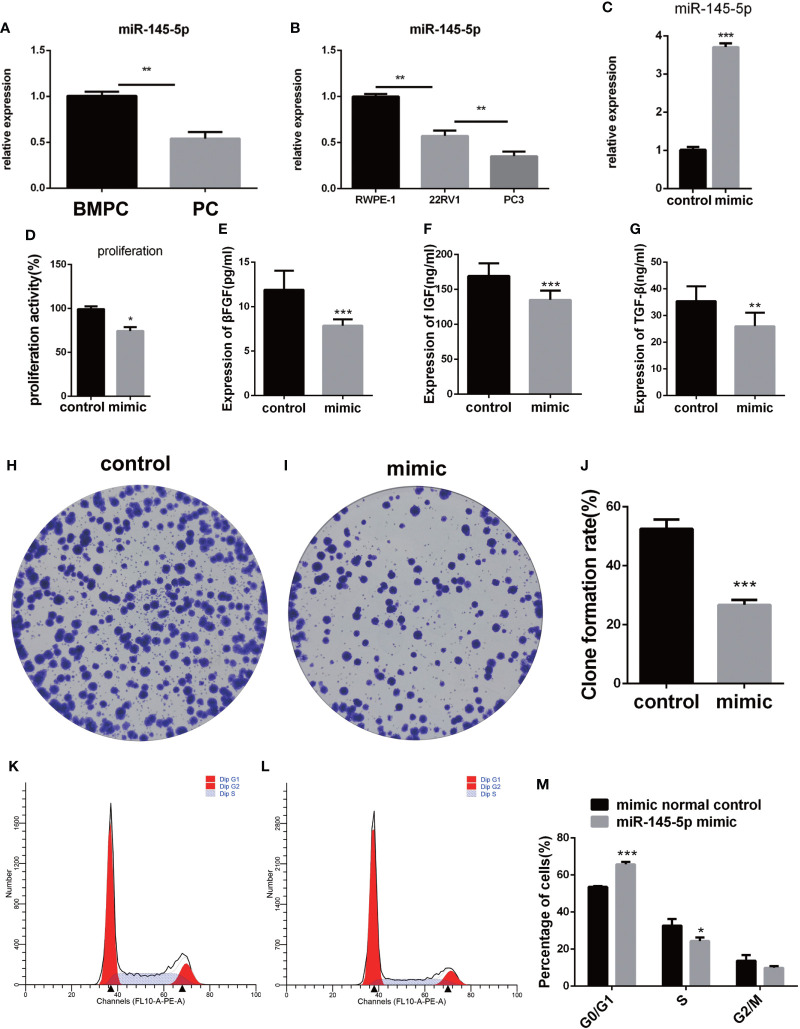
miRNA-145-5p inhibited proliferation of bone metastatic Pca cells, PC-3. **(A)** RT-qPCR data for miRNA-145-5p expression in Pca bone metastases. **(B)** RT-qPCR data for miRNA-145-5p expression in PC3, 22RV1 and RWPE-1 cell lines. **(C)** miRNA-145-5p expression after PC3 transfection. **(D)** Proliferation rate of PC3 cells. **(E–G)** Expression of βFGF, IGF and TGF-β in PC3 cells. **(H–J)** Clone formation rate of PC3 cells. **(K–M)** Flow cytometry cell cycle analysis of PC3 cells. *P < 0.05, **P < 0.01, ***P < 0.001.

miRNA-145-5p expression was detected by RT-qPCR and showed significant decreases in Pca tissues with bone metastasis compared with those without metastasis (**Figure 3A**). The metastatic Pca cell line, PC3, expressed lower levels of miRNA-145-5p than the non-metastatic, 22RV1, or non-cancerous prostate epithelial, RWPE-1, cell-lines (**Figure 3B**). PC3 cells were transfected with an exogenous miRNA-145-5p mimic or control miRNA and expression of miRNA-145-5p shown to be increased by RT-qPCR in transfected cells compared with controls (**Figure 3C**). PC3 cells with higher miRNA-145-5p expression had reduced rates of proliferation, measured by CCK-8 and clone assay (**Figure 3D** and **Figures 3H** **–** **J**). Higher miRNA-145-5p expression also lengthened the G1 phase to slow proliferation (flow cytometry results shown in **Figures 3K** **–** **M**). Higher miRNA-145-5p expression attenuated the production of bFGF, IGF and TGF-β proteins in PC3 cells, as determined by ELISAs (**Figures 3E** **–** **G**). Thus, miRNA-145-5p changed the protein expression profile and influenced the cell cycle with the result that PC3 cells became less proliferative.

### miRNA-145-5p reduced invasion and migration by regulating the EMT

**Figure 4:**
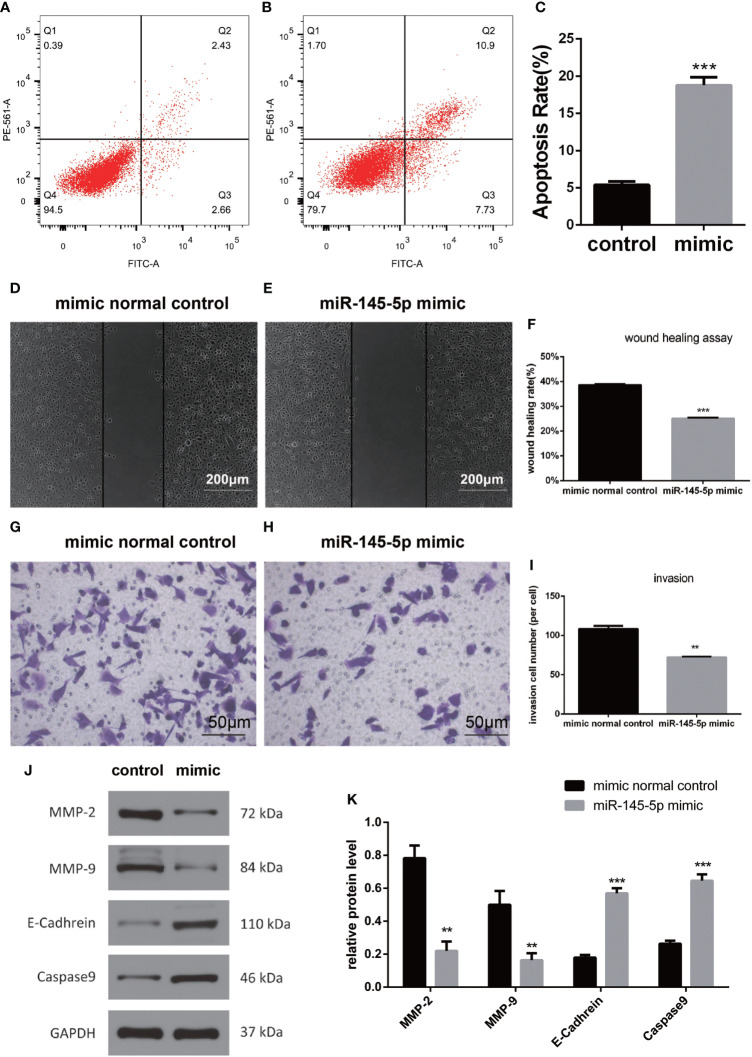
miRNA-145-5p affected the EMT to inhibit invasion and migration in bone metastatic Pca cells. **(A, B)** Flow cytometry cell apoptosis analysis of PC3 cells. Cells are distributed differently throughout the four quadrants of the plot depending on the degree of apoptosis undergone by cell population. Apoptotic cells appear in the greatest concentration in quadrant, Q2. The percentage of cells in Q2 relative to the total population of cells is shown in **(C)**. **(D–F)** Scratch tests of PC3 cell migration following miRNA-145-5p mimic transfection. (mean ± SEM, n = 3, Student’s t-test). **(G–I)** Transwell results of PC3 cell invasions after miRNA-145-5p mimic transfection. **(J, K)** Caspase 9, E-cadherin, MMP-2 and MMP-9 protein expression in PC3 cell line after miRNA-145-5p mimic transfection. **P < 0.01, ***P < 0.001.

**Figure 5:**
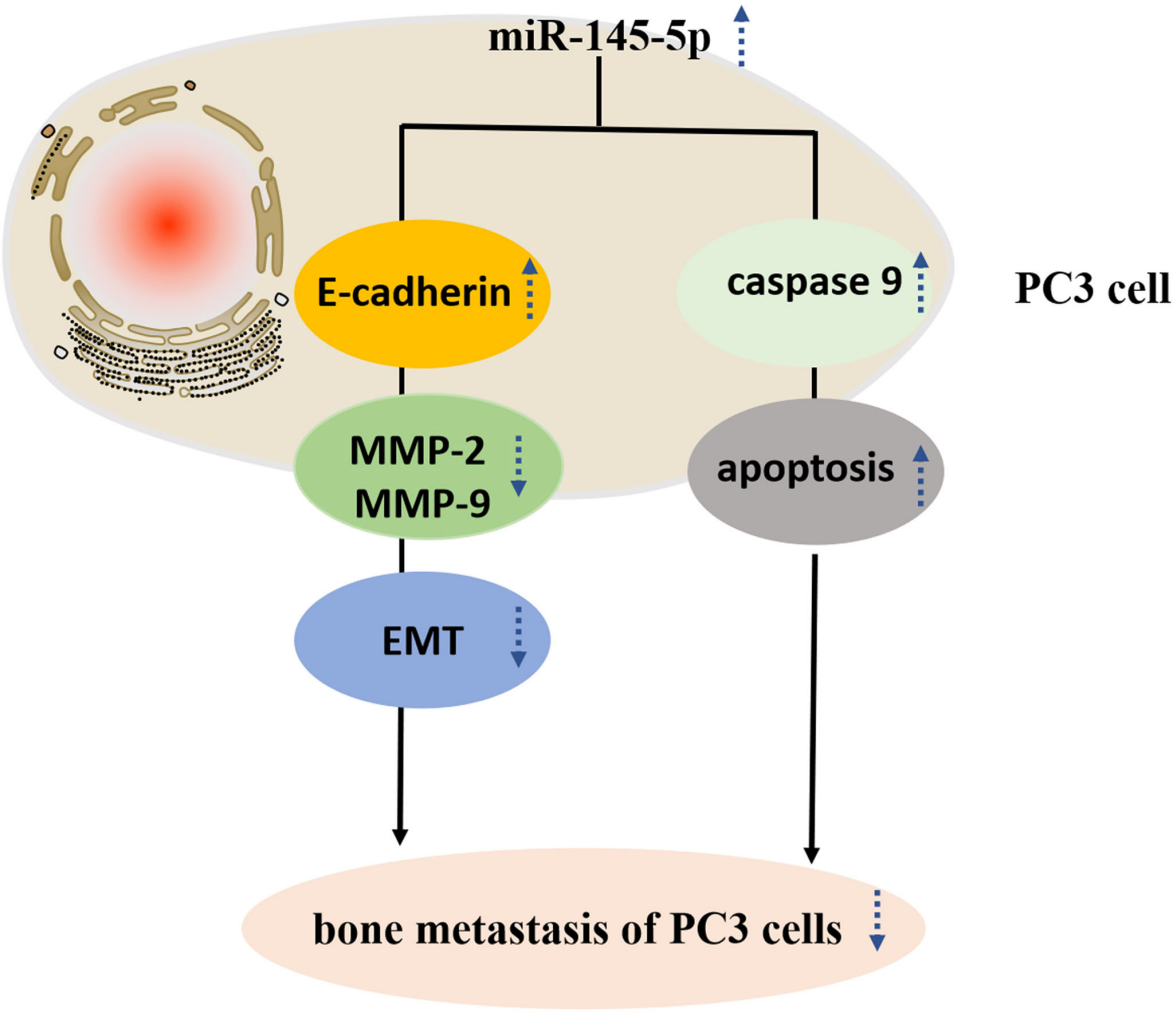
Study design flowchart.

Assessments of PC3 cells apoptosis demonstrated that miRNA-145-5p expedited this process (**Figures 4A–C**). Scratch and Transwell assays showed that transfection with the miRNA-145-5p mimic reduced PC3 cells migration and invasion (**Figures 4D** **–** **I**). Furthermore, upregulation of miRNA-145-5p enhanced expression of E-cadherin (an epithelial marker), and decreased expression of MMP-2 and MMP-9 (mesenchymal markers) as measured by Western blotting (**Figures 4J**, **K**). Moreover, miRNA-145-5p activated the apoptosis-associated protein, caspase 9, implying the promotion of programmed cell death in PC3 cells. In summary, miRNA-145-5p appeared to enhance the expression of epithelial marker (E-cadherin) while suppressing expression of mesenchymal markers (MMP-2 and MMP-9), implying a regulatory action on the epithelial to mesenchymal transition. Increased expression of caspase-9, an enzyme associated with apoptosis, implies that increased expression of miRNA-145-5p may act to induce apoptosis of bone metastatic Pca cells (**Figure 5**).

## Discussion

Pca patients with bone metastasis may suffer severe complications, including severe back and extremity pain, pathological fracture, deep vein thrombosis and urinary tract infection (14–16). The prognosis for these patients remains poor and more advanced treatment strategies are needed. miRNAs have great versatility and have been incorporated into various cancer treatments to enhance specificity and therapeutic efficacy (8). The current study identified miRNAs that were differentially expressed when Pca metastasized into bone and indicated the potential for the development of the miRNA-145-5p mimic into a novel treatment for metastatic Pca.

Ozen *et al.* reported the SOX-2 regulatory activity of miRNA-145-5p which inhibited Pca cell proliferation. The current study found low levels of miRNA-145-5p expression in Pca bone metastatic tissues and cell lines. Upregulation of its expression suppressed the migratory and invasive behavior of the cells. It has previously been found that miRNA-145-5p attenuated the invasive properties of glioblastoma cells and reduced osteosarcoma progression (17, 18). The target gene of miRNA-145-5p was associated with the TGF-β signaling pathway, a multi-functional regulatory pathway responsible for cell proliferation, migration, apoptosis and adhesion (19). TGF-β signaling has tumor suppressive functions in untransformed and early cancer cells in which it promotes cell cycle arrest and apoptosis (19). However, its activation in advanced cancer promotes tumorigenesis, including metastasis and drug resistance. The dual function and pleiotropy of TGF-β signaling make it a challenging therapeutic target (20). Other growth factors were also found to be altered in expression during the current study. Increased bFGF expression has previously been shown to stimulate osteoclastogenesis, inducing bone metastasis in Pca (21). In addition, high levels of IGF in the primary tumor environment may induce tumor cells to metastasize to bone (22). Prostate tumors within bone tissue promote osteoclast-mediated bone resorption to release TGF-β. Blocking TGF-β may thus improve patient survival (23). In this study, TGF-β2 was predicted using miRNA-TarBase as a target gene of miR-145-5p. The current findings regarding reduced bFGF, IGF and TGF-β expression on upregulation of miRNA-145-5p indicate that miR-145-5p may influence the TGF-β signaling pathway by suppressing epithelial to mesenchymal transition and tumor growth to bring about inhibition of bone metastasis.

The EMT involves the loss of epithelial phenotypic characteristics and adoption of those of the mesenchymal phenotype, including enhanced migratory and invasive properties. Overactivation of the transition is considered to promote cancer metastasis (24). Cancer cell invasion and metastasis is assisted by activities of MMP-2 and MMP-9 which are associated with promoting cell-cell and cell-matrix interactions during the EMT (25). The current study demonstrated that MMP-2 and MMP-9 expression were reduced by miRNA-145-5p. Loss of the tumor suppressor, E-cadherin, has frequently been related to tumor metastasis and its expression was shown to be stimulated by miRNA-145-5p during the present study (26, 27). EMT master genes transcription factors include SLUG, SNAIL, ZEB1, ZEB2/SIP1, TWIST1, TWIST2, the homeobox proteins (GSC and SIX1) and the forkhead-box protein FOXC2, which function as direct or indirect suppressors of the E-cadherin (epithelial marker) and inducers of MMP2 and MMP9 (mesenchymal markers) (27). This study indicated that miR-145-5p might be transcriptionally regulated by EMT master genes transcription factors. Caspase 9 is noteworthy as a promoter of apoptosis and its expression was also stimulated by miRNA-145-5p (28). In summary, we find that miRNA-145-5p reduces makers of migration and invasiveness and stimulates tumor suppressor and apoptosis stimulatory proteins in PC3 cells. These effects combine to indicate an anti-metastatic action for miRNA-145-5p. Moreover, previous work has established reduced miRNA-145-5p expression in the apocrine and spindle cells of transforming tumor cells compared with *in situ* carcinoma or nontumor structures which further substantiates the role of miRNA-145-5p in regulating metastasis (29). RNA-based therapeutics, including miRNAs, messenger RNAs (mRNAs), antisense oligonucleotides (ASOs), small interfering RNAs (siRNAs) and aptamers, are a rapidly developing new field with inspiring potential (30–32). However, RNA-based therapeutic drug delivery remain critical hurdles. Lipid-based nanoparticle delivery systems, containing a variety of effective RNA-base therapeutic payloads, can ensure specific delivery of therapeutic drugs to tissues and exert attractive properties (31–33). From the part of the clinical trials, RNA-based therapeutics will provide a promising treatment approach for various human diseases.

## Conclusions

In summary, profiles of miRNAs differentially expressed in Pca with and without bone metastasis were characterized. This study revealed that miR‐145‐5p was a negative regulator of EMT and promoted apoptosis in Pca bone metastasis. Moreover, over-expression of miRNA-145-5p reduced invasiveness and proliferation in prostate cancer bone metastatic cell-line. We suggest that mimicry of miRNA-145-5p may constitute a novel target in the treatment of Pca bone metastasis.

## Data availability statement

The datasets presented in this study can be found in online repositories. The names of the repository/repositories and accession number(s) can be found in the article/**Supplementary Material**.

## Ethics statement

The studies involving human participants were reviewed and approved by The University of Hong Kong-Shenzhen Hospital Research Ethics Committee approved this research (No. [2019]260). The patients/participants provided their written informed consent to participate in this study. Written informed consent was obtained from the individual(s), and minor(s)’ legal guardian/next of kin, for the publication of any potentially identifiable images or data included in this article.

## Author contributions

BL and YY conceived and designed the project. BL wrote the paper. YZ, SL, RD, JH, PL, and YX acquired the data. G-MK and ZL analyzed and interpreted the data. RL modified the language of the article. All authors contributed to the article and approved the submitted version.

## Funding

This research is supported by the project “High Level Hospital Program, Health Commission of Guangdong Province, China” (No. HKUSZH201901024 and No. HKUSZH201901012) and Research project of Health Commission of Guangdong Province, China (B2021176). The funders had no role in study design, data collection and analysis, decision to publish,or preparation of the manuscript.

## Conflict of interest

The authors declare that the research was conducted in the absence of any commercial or financial relationships that could be construed as a potential conflict of interest.

## Publisher’s note

All claims expressed in this article are solely those of the authors and do not necessarily represent those of their affiliated organizations, or those of the publisher, the editors and the reviewers. Any product that may be evaluated in this article, or claim that may be made by its manufacturer, is not guaranteed or endorsed by the publisher.

## Supplementary material

Supplementary Table 1 A List of differentially expressed genes associated with bone metastases from prostate cancer.

Supplementary Table 2 KEGG analysis of miR-145-5p target genes.
